# Comparison of Intravoxel Incoherent Motion Diffusion-Weighted MR Imaging and Arterial Spin Labeling MR Imaging in Gliomas

**DOI:** 10.1155/2015/234245

**Published:** 2015-04-05

**Authors:** Yuankai Lin, Jianrui Li, Zhiqiang Zhang, Qiang Xu, Zhenyu Zhou, Zhongping Zhang, Yong Zhang, Zongjun Zhang

**Affiliations:** ^1^Department of Medical Imaging, Jinling Hospital, Medical School of Nanjing University, Nanjing 210002, China; ^2^School of Medical Imaging, Xuzhou Medical College, Xuzhou 221000, China; ^3^GE Healthcare China, Beijing 100000, China

## Abstract

Gliomas grading is important for treatment plan; we aimed to investigate the application of intravoxel incoherent motion (IVIM) diffusion-weighted imaging (DWI) in gliomas grading, by comparing with the three-dimensional pseudocontinuous arterial spin labeling (3D pCASL). 24 patients (13 high grade gliomas and 11 low grade gliomas) underwent IVIM DWI and 3D pCASL imaging before operation; maps of fast diffusion coefficient (*D* ^∗^), slow diffusion coefficient (*D*), fractional perfusion-related volume (*f*), and apparent diffusion coefficient (ADC) as well as cerebral blood flow (CBF) were calculated and then coregistered to generate the corresponding parameter values. We found CBF and *D* ^∗^ were higher in the high grade gliomas, whereas ADC, *D*, and *f* were lower (all *P* < 0.05). In differentiating the high from low grade gliomas, the maximum areas under the curves (AUC) of *D* ^∗^, CBF, and ADC were 0.857, 0.85, and 0.902, respectively. CBF was negatively correlated with *f* in tumor (*r* = −0.619, *P* = 0.001). ADC was positively correlated with *D* in both tumor and white matter (*r* = 0.887, *P* = 0.000 and *r* = 0.824, *P* = 0.000, resp.). There was no correlation between CBF and *D* ^∗^ in both tumor and white matter (*P* > 0.05). IVIM DWI showed more efficiency than 3D pCASL but less validity than conventional DWI in differentiating the high from low grade gliomas.

## 1. Introduction

Gliomas are the most common primary tumor in brain. More accurate preoperative grading of gliomas could increase the rationality of treatment plans and judge prognosis. Generally, blood supply of high grade gliomas is significantly higher than that of low grade gliomas. Perfusion imaging can assess tumor blood supply, which could provide a reference for glioma grading. The main nonenhanced method of perfusion in MRI is arterial spin labeling (ASL). In addition, there is another solution, intravoxel incoherent motion (IVIM); it also does not need contrast agent; thus it is safe, repeatable, and without radiation burden.

IVIM is the microscopic translational movement occurring in each image voxel during an MRI acquisition, which was proposed by Le Bihan et al. [1] in 1988. In the horizontal voxel size, capillary distribution can be considered as isotropic and random; therefore, the water molecule movement in the capillary also can be considered as an incoherent motion. By applying a diffusion gradient field of high *b* values, the biexponential model can differentiate the diffusion in extracellular from that in intracellular, which are defined as fast diffusion coefficient *D* ^∗^ and slow diffusion coefficient *D*, respectively. The perfusion fraction *f* can be obtained simultaneously, which describes the fraction of incoherent signals which come from the vascular component [2]. The IVIM had been used for many tumors cases in recent years, such as hepatic carcinoma [3], renal cancer [4], prostate cancer [5], and salivary gland tumors [6], while less had been done for IVIM at gliomas [7–9]. With more and higher *b* values, IVIM biexponential model could better demonstrate the decay curves of diffusion signal in brain gray and white matters [2, 10, 11]. However, in previous studies about gliomas, the maximum and number of *b* value in DWI were relatively low and small (1,300 s/mm^2^ and 14 in [7], 900 s/mm^2^ and 16 in [8], and 3,500 s/mm^2^ and 13 in [9]), which might affect the accuracy of the outcomes. Besides, the signal-to-noise ratio (SNR) of IVIM images in these studies was limited.

The 3D pCASL is one submethod of ASL. It had been widely studied in gliomas and had reliable results with dynamic susceptibility contrast (DSC) [12–14]. The purpose of this study was to use the IVIM biexponential model based on DWI with higher (up to 3,500 s/mm^2^) and more (20) *b* values to analyze the characteristics of gliomas and to compare with the 3D pCASL findings.

## 2. Materials and Methods

### 2.1. Subjects

The present study was approved by the Local Institutional Review and a written informed consent was obtained from all patients. From September 2013 to June 2014, 24 patients (nineteen men and five women, age range from 16 to 65 years, mean age 42 ± 14.24 years) confirmed as having gliomas by pathology were recruited and took MRI examinations before operation. The pathology results of all subjects were shown in Table 1.

### 2.2. Imaging Parameter

All MR data was acquired on a 3 T magnetic resonance (MR) scanner (Discovery MR750 System; GE Medical Systems, Milwaukee, WI, USA) with an 8-channel receiver head coil. DW-MR imaging was based on a standard Stejskal-Tanner diffusion-weighted spin-echo EPI pulse sequence with the following parameters: TR/TE = 3000/87.5 ms, field of view (FOV) = 24.0 cm, base resolution = 128 × 128, slice thickness = 5.0 mm, intersection gap = 1.5 mm, and a bandwidth = 250 Hz. Axial DW-imaging was acquired with multiple *b* values ranging from 0 s/mm^2^ to 3,500 s/mm^2^ (i.e., 0, 10, 20, 40, 80, 110, 140, 170, 200, 300, 400, 500, 600, 700, 800, 900, 1,000, 2,000, 3,000, and 3,500 s/mm^2^) in three orthogonal directions. With the increase of *b* values, the number of excitations (NEX) also increased from one to six to ensure a good SNR; the acquisition time was last 330 s. ASL perfusion imaging was performed with pseudocontinuous labeling, background suppression, and a stack of spirals of 3D fast spin-echo imaging sequences. Images were acquired with the following parameters: 512 sampling points on eight spirals, TR/TE = 5327/10.5 ms, postlabel delay (PLD) = 1.5 s, FOV = 24.0 cm, bandwidth = ±62.5 KHz, slice thickness = 4.0 mm, number of slices = 36, NEX = 3.0, and an acquisition time of 309 s.

After acquiring DWI and ASL imaging, axial T2-weighted imaging (Propeller, TR/TE = 4,300/103 ms, slices with thickness = 5.0 mm, and intersection gap = 1.5 mm), 3D T1-weighted imaging (3D-T1WI, BRAVO, TR/TE = 8,200/3,200 ms, slice thickness = 4.0 mm, and number of slices = 36), and coronal T2-fluid attenuated inversion recovery weighted imaging (TR/TE = 4,300/93 ms, slices with thickness = 5.0 mm, and intersection gap = 1.5 mm) were acquired. Axial T1-weighted imaging (FLAIR, TR/TE = 1,750/24 ms, slices with thickness = 5.0 mm, and intersection gap = 1.5 mm) was acquired before and after intravenous body weight adapted administration of gadobutrol (Dextran 40 Glucose Injection, Consun Pharmaceutical Group, Guangzhou, Guangdong, China).

### 2.3. Postprocessing of DWI and ASL Imaging

The DWI imaging was calculated by at two-segment monoexponential algorithm as shown in IVIM equation (1), where *D* and *D* ^∗^ are the diffusion parameters related to molecular diffusion and to the perfusion-related diffusion, respectively, *S*/*S*0 is the normalized signal attenuation, and *f* is the perfusion fraction. Setting 200 s/mm^2^ as the cutoff of the low *b* values, the high values will generate the *D* first, and the low *b* values will yield the *D* ^∗^ and *f* at the same time after removing the effects of *D*, finally producing the *D*, *D* ^∗^, and *f* maps. ADC map was also calculated by *b* values of 0 and 1000 s/mm^2^ with monoexponential equation (2). ASL imaging was analyzed by corresponding software to produce CBF maps. All processes were calculated by GE AW4.6 workstation automatically:

(1) $$\begin{array}{c} \frac{S}{S0}=\left(1-f\right)\cdot \exp\left(-bD\right)+f\cdot \exp\left(-{bD}^{*}\right), \end{array}$$

(2) $$\begin{array}{c} \frac{S}{S0}=\exp\left(-bD\right). \end{array}$$

### 2.4. Image Coregistration and Regions of Interest (ROIs)

**Figure 1:**
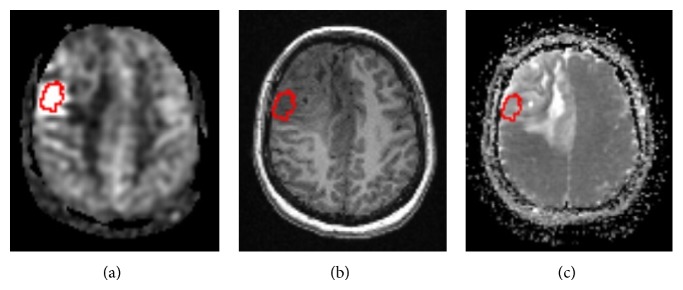
Coregistered images with ROIs. CBF map (a) and corresponding slice of 3D-T1WI (b) as well as DWI map (c).

Diffusion maps and structural 3D-T1WI images and were, respectively, coregistered to CBF images using SPM8 (http://www.fil.ion.ucl.ac.uk/spm/) by applying rigid-body transformations. And then, these maps were resliced to match the CBF maps voxel-by-voxel [15]. ROIs were drawn in the regions of tumors whose blood supplies were the richest as solid component as well as normal white matter in contralateral semioval center in the CBF maps, combined with the coregistered 3D-T1WI slices to avoid cystic, hemorrhage, necrosis, and ischemic area; then the ROIs were extracted to cover the coregistered DWI maps, using MRIcroN (http://www.mccauslandcenter.sc.edu/mricro/), Figure 1. Perfusion and diffusion data from the ROIs were analyzed by Matlab (MathWorks, Natick, MA) to get median, mean, standard deviation, kurtosis, skewness, maximum, minimum, and the 90th percentile of all voxels to make a histogram analysis such as [15].

### 2.5. Statistical Analysis

Normal probability plot and Shapiro-Wilk's test were used to analyze data distribution. Pairwise comparisons of ASL and IVIM parameters between HGG and LGG were calculated. Bivariate correlations of parameters of ASL and IVIM DWI as well as IVIM DWI and conventional DWI were assessed. Student's* t*-test and Pearson test were used for normally distributed data, and a nonparametric test (Mann-Whitney *U*) and Spearman test applied for data that did not fulfill the requirements for normality. Differences in all parameters between HGG and LGG gliomas were further analyzed by using receiver-operating characteristic (ROC) curves and comparing the area under the curve (AUC). The parameter with the highest AUC was chosen as the best discriminating parameter. All analyses were performed by SPSS Version 16.0 for Windows (SPSS, Chicago); a *P* < 0.05 was considered as statistical significance.

## 3. Results

### 3.1. ASL and IVIM Imaging

**Figure 2:**
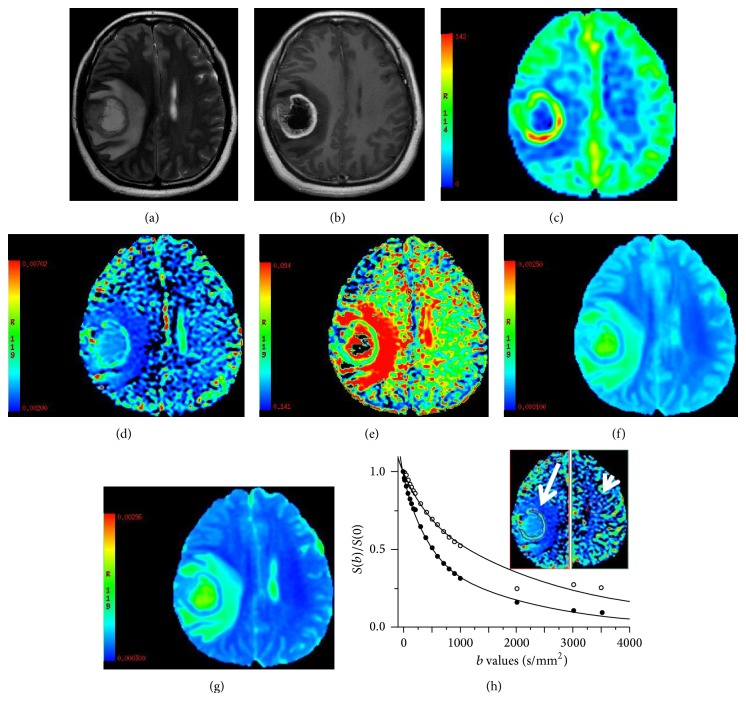
Female, aged 43 years with a glioblastoma of the right parietal lobe (WHO IV). The axial T2-weighted image (a) shows the medium signal thick walled mass with a hyperintense central cystic necrosis and peritumoral edema. Enhanced T1-weighted images (b) show the significantly enhanced tumor wall. CBF map (c) shows that the wall of the mass with unevenly high perfusion; the TBF and CBF of contralateral semioval center were 89.90 and 16.89 mL/100 g/min, respectively. The *D* ^∗^ map (d) demonstrates the increased fast diffusion values in the tumor tissue and shows good agreement with the CBF map in the area of high perfusion tumor tissue and low perfusion peritumoral edema. The *f* map (e) demonstrates the gadolinium enhancing region of the tumor and exhibits the edge of the tumor more clearly. The *D* map (f) fails to delineate the tumor clearly but better describes the scope of peritumoral edema. The ADC map (g) was similar to the *D* map in showing the tumor. The *D* ^∗^, *D*, *f*, and ADC value of the tumor and the contralateral semioval center were *D* ^∗^, 3.01, 2.37 × 10^−3^ mm^2^/s; *D*, 0.61, 0.36 × 10^−3^ mm^2^/s; ADC, 0.779, 0.456 × 10^−3^ mm^2^/s; *f*, 0.42, 0.26. The logarithmic plot of signal intensity decays as a function of *b* with a corresponding biexponential fit (h): the mass (arrow and solid circles) and contralateral semioval center (arrow head and hollow circles).

**Figure 3:**
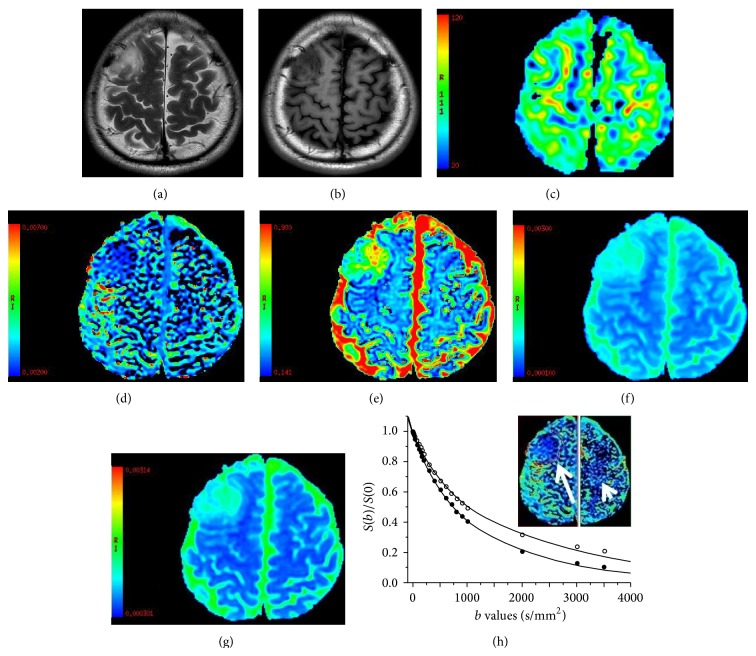
Male, aged 33 years with astrocytoma glioma in the right parietal (WHO II). The mass was hyperintensive with a fuzzy edge in axial T2-weighted image (a) and without any gadolinium enhancement (b). It shows slight perfusion in the CBF map (c) but fails to distinguish the boundaries of the tumor from normal brain substance. The TBF and CBF of contralateral semioval center were 80.72 and 27.53 mL/100 g/min, respectively. The *D* ^∗^ map (d) and *f* map (e) well demonstrate the borderlines of the tumor as an enhancement image. The *f* map also shows an inhomogeneous intrinsic hyperintensity in the tumor. The *D* map (f) fails to delineate internal details of the tumor but better describes the scope of peritumoral edema. The ADC map (g) resembled *D* map. The *D* ^∗^, *f*, and *D* value of tumor and contralateral healthy semioval center were *D* ^∗^, 2.73, 2.30 × 10^−3^ mm^2^/s; *D*, 0.74, 0.41 × 10^−3^ mm^2^/s; ADC, 0.868, 0.524 × 10^−3^ mm^2^/s; *f*, 34.65%, 0.28, respectively. The logarithmic plot of signal intensity decay as function of *b* with corresponding biexponential fit (h) of the tumor (arrow and solid circles) and contralateral semioval center (arrow head and hollow circles).

The ASL and IVIM-DWI data of 24 patients yielded high quality images with good SNR with CBF and IVIM postprocessing (Figures 2(c)–2(g) and 3(c)–3(g)). The curves calculated by biexponential model fit well with the signal attenuation characteristics with the increase of *b* values in this study; setting contralateral semioval center as the reference, signal attenuation of tumor tissue was more rapid, especially in high grade gliomas (Figures 2(h) and 3(h)).

### 3.2. Mean ASL and IVIM Parameters in HGG as well as LGG

Mean CBF and *D* ^∗^ were higher in the HGG (*P* < 0.05), whereas mean *f*, ADC, and *D* tended to be lower (the former one *P* < 0.05, the following two *P* < 0.01). There were almost no differences between contralateral semioval center of two groups in all parameters mentioned above (all *P* > 0.05), Table 2.

### 3.3. Correlation Analysis

In tumors, CBF was negatively correlated with the *f* (*r* = −0.619, *P* = 0.001). ADC was strongly positively correlated with *D* in both tumor and white matter (*r* = 0.887, *P* = 0.000 in former and *r* = 0.824, *P* = 0.000 in latter). There was no correlation between CBF and *D* ^∗^ in both tumor and white matter (both *P* > 0.05). All the correlation analyses above were done by Spearman test.

### 3.4. Histogram and ROC Analysis of Perfusion and Diffusion Parameters

Histogram parameters in tumor from the ASL and IVIM DWI scans were compared between HGG and LGG. For ASL, the maximum of CBF had the lowest *P* value (0.002) and biggest AUC (0.85) for separating HGG and LGG. For IVIM DWI, the mean of *D* ^∗^ had lowest *P* value (0.002) and biggest AUC (0.857) for discriminating HGG and LGG, while, in conventional DWI, the median of ADC showed the lowest *P* value (0.000) and best AUC (0.92) in all parameters mentioned above for distinguishing HGG and LGG, Table 3. Histogram analysis showed almost no significant difference of all parameters of white matter between HGG and LGG, Table 4.

## 4. Discussion

There were many kinds of water molecule movements in brain tissue, including intracellular, intercellular, and transmembrane molecular diffusion, as well as microcirculation of blood in the capillary network. It has been confirmed that when applying a diffusion gradient field of high *b* values, signal attenuation of water molecule diffusion in brain tissue is following the way of multiexponential models, which (multiexponential models) can better reflect the actual diffusion information of brain parenchyma than monoexponential model [16]. Compared to the multiexponential model, the biexponential model might be oversimplified, but in regard to the evaluation of two or more different proton diffusion pools in voxel, the biexponential model is more feasible [2, 17]. Federau et al. [18] found that IVIM can monitor the change in cerebral blood flow caused by the expansion or contraction induced by CO_2_ and O_2_. Wirestam et al. [19] indicated that *f* and CBF derived from IVIM agree reasonably with conventional CBV and CBF get from DSC, respectively. The studies mentioned above revealed that it was feasible to evaluate the perfusion of brain tissue by IVIM. Once more, the applications of IVIM in varieties of pathological changes in brain tissue, such as stroke, gliomas, metastasis, and encephalitis, also show the potential advantages of IVIM in discovering the abnormal brain tissue [7–9].

Compared to previously recent published works in gliomas [7–9], in this research more and greater *b* values were used, as more *b* values in segment of low *b* values can get more acute perfusion-related diffusion, while higher *b* values can better eliminate the perfusion-related diffusion; thus it can in turn generate a more realistic molecular diffusion coefficient value and perfusion-related diffusion [2, 10, 11]. A histogram analysis of data also allows for a deeper analysis of the perfusion or diffusion distribution in the tumor that exceeds that of mean value.

For example, mean values of *D* and ADC had closed AUC in identification of LGG from HGG; however, the median value of ADC showed the lower *P* value and better AUC than the former two.

Like CBF, *D* ^∗^ of HGG was significantly higher than that of LGG in this study; mean of *D* ^∗^ was the most efficient in differentiating the HGG from LGG in three parameters of IVIM DWI. As *D* ^∗^ values could indirectly reflect tumor tissue perfusion, this result met the perfusion characteristics of gliomas, although *D* ^∗^ does not have a direct correlation with CBF in this study. Compared to the results obtained by Bisdas et al. [7], the *D* ^∗^ value in this study was relatively small, probably due to the fact that the higher *b* values were used. The larger *b* value, the more signal attenuation, which may affect the results as well as different fitting algorithms that can also be seen in [9]. In the *D* ^∗^ map, it was found that the *D* ^∗^ value for the tumors varied widely, perhaps because of the greater heterogeneity of tumor tissue, as reported by Bennett et al. [20].

In this study, the *f* value of high grade gliomas was lower than that of low grade gliomas, which was not consistent with [7]. The correlation analysis also found that the *f* value of the tumor was negatively correlated with TBF. As the *f* value is the volume fraction of the rapid diffusion component in voxel, it could also reflect the perfusion of tumor tissue. So the *f* value should be higher in high grade gliomas and positively correlated with CBF theoretically. In addition to the variation among selected tumor cases and structural characteristics of tumors, the different choices of *b* values and parameters of DWI scheme might also be the reasons that led to the opposite result, according to Guiu et al. [21] and Lemke [22]. Time of echo (TE) is an independent factor of *f*; the longer the TE, the more signal attenuation at low *b* values, and the greater *f* value. Although Bisdas et al. [7] reported that the *f* value was higher in high grade gliomas than that in low grade gliomas, their results also showed that the average *f* value of white matter was higher than that of low grade gliomas and the similar results also can be seen in [9], so the impact coming from TE on *f* value of different brain tissues still needs further study. We also found the *f* values of lateral ventricles and edema around the tumors are large, which means the *f* value is easy polluted by cerebrospinal fluid and edema. For these reasons, before setting a proper TE and removing the effects of cerebrospinal fluid and edema, an accurate *f* value of tumor may not be acquired. Nonetheless, the *f* map shows a high contrast in the scope and details of the tumor and may have extensive application.

Compared to LGG, the *D* value tended to be lower in HGG in this study. As *D* mainly reflects the Brownian motion of water molecules in the organization, which is more limited by smaller intercellular gaps, it is in line with the characteristic of gliomas, that is, the higher the grade, the greater the cell density. This is also consistent with the result of IVIM research in gliomas [9] and prostate cancer [5]. Compared to [7–9], the efficacy of *D* in identifying HGG from LGG was lower than that of ADC derived from only two *b* values (0 s/mm^2^ and 1,000 s/mm^2^) in this study, but there is a strong correlation between *D* and ADC in both tumor and white matter, and *D* map was comparable with ADC map in the exhibition of the scope and detail of tumor, whether *D* could replace the ADC is still worthy of further study.

Through the measurement process of the cerebral cortex, it was found that the cerebrospinal fluid in sulcus had a larger impact on the *D* ^∗^ and *f* value of cerebral cortex, which is consistent with the literatures [7, 8]. This is because there are two kinds of water molecules movements in cerebrospinal fluid, that is, free diffusion and liquidity. Small blood vessels in sulcus also affect the results of the cortex in IVIM or ASL images. AS IVIM and ASL were based on mathematical models, IVIM results were influenced by the distribution of *b* values and parameters of DWI [11, 22]. The positions of subjects and PLD will affect the results of ASL [23, 24]. And the average age of patients with HGG was greater than that of patients with LGG in this study. All these aforementioned factors will affect the results.

Currently, perfusion methods of MRI were mainly DSC and ASL. DSC was based on precise understanding of the arterial input function and a lot of assumptions. Thus, it was difficult to measure and quantify [25]. When applied to the brain, its low SNR, partial volume effects, susceptibility artifacts, and other factors outside the brain such as heart rate, cardiac output, and arterial stenosis would affect the results [26, 27]. When DSC was applied to a brain tumor, there were also deviations caused by leaking contrast agent and vascular contamination. Patients with radiotherapy-induced vasculitis could not accept the bolus-injection pressure and the rate of high-pressure syringe. Contrast agent also may cause nephrogenic systemic fibrosis in patients with renal insufficiency. The mechanism of ASL uses the protons of freely diffused water molecules in the artery as an endogenous tracer, making subtraction between the before and after marking images, thus obtaining subtraction images only with perfusion information. In the absence of impacts of exogenous contrast agents, the physical and chemical characteristics of the blood are unchanged, while influence of blood-brain barrier damage is also avoided. For some perspective, ASL could better reflect the hemodynamic parameters of the issue itself and has less susceptibility to any artifacts [12–14]. It may be more valuable in those diseases, which could lead to damage of blood-brain barrier, such as malignant gliomas. However, the SNR of ASL is low, and as described above, results of ASL are sensitive to the subjects' postures and instrument parameters, and so forth.

Like ASL, IVIM does not need a contrast agent. It is based on the characteristics of the tissue itself. It stimulates and reads the information in voxels successively, and it is mainly sensitive to the increase of incoherent motion in voxel. Therefore, it is not directly affected by the anterior cerebral vascular (e.g., stenosis of internal carotid artery or vertebral artery), or the cardiac output and subjects' postures. As Henkelman [28] pointed out, classical perfusion measures the pattern of delivery, while IVIM measures the traffic flow in the direction of encoding gradient. The link between IVIM and classical perfusion had been discussed in detail [29]. For these reasons, IVIM could be a supplemental perfusion method to conventional perfusion sequences, but the influence of DWI parameters on the accuracy of IVIM parameters and cerebrospinal fluid contamination still needs further study.

## 5. Conclusion

The IVIM DWI shows efficacy in differentiating the low grade from high grade gliomas, *D* ^∗^ was better than CBF calculated by 3D pCASL, perfusion-related parameter *f* was negatively correlated with the CBF, and *f* map could show more clear scope of tumor. Though the efficacy of *D* in identifying HGG from LGG was lower than ADC derived from conventional DWI, there was a strong correlation between them. Limitation of this study was that the sample size was small, and the subjects' ages in high grade gliomas group were considerably higher than those in low grade gliomas group. In summary, IVIM MR imaging could get the perfusion and diffusion information of gliomas simultaneously and might enable a noninvasive method in differentiating grade LGG from HGG gliomas.

## Figures and Tables

**Table 1:** Summary of pathology of all subjects.

	WHO grade	Age	Pathology	Location
HGG	WHO III-IV *N* = 13	Average, 51 years Range, 38–65 years	5 glioblastomas	3 in parietal lobe 2 in temporal lobe
			8 anaplastic astrocytomas	4 in the frontal lobe
				1 in parietal lobe
				1 in temporal lobe
				1 in lateral ventricle
				1 in vermis cerebella

			7 diffuse astrocytomas	3 in temporal lobe 4 in frontal lobe
LGG	WHO I-II *N* = 11	Average, 32 years Range, 16–53 years	3 oligodendrogliomas	2 in the parietal lobe 1 in the lateral ventricle
			1 capillary astrocytoma	1 in cerebellar

HGG: high grade gliomas, LGG: low grade gliomas.

**Table 2:** Mean ASL and IVIM parameters in HGG and LGG as well as ROC analysis.

		HGG	LGG	Test type	*P* value	AUC	Cutoff value
Tumor	TBF	102.04 ± 45.55	62.26 ± 18.13	tt	**0.011**	0.783	63.17
	*D* ^∗^	5.1 ± 3.39	2.77 ± 0.69	mw	**0.002**	0.857	3.5
	*D*	0.69 ± 0.09	0.81 ± 0.12	tt	**0.013**	0.818	0.58
	*f*	0.4 ± 0.11	0.49 ± 0.1	tt	**0.039**	0.769	0.43
	ADC	0.85 ± 0.09	1.04 ± 0.13	tt	**0.001**	0.85	0.96

WM	CBF	25.25 ± 3.46	27.64 ± 8.21	mw	0.733		
	*D* ^∗^	2.5 ± 0.18	2.52 ± 0.29	mw	0.691		
	*D*	0.41 ± 0.02	0.41 ± 0.02	tt	0.801		
	*f*	0.32 ± 0.03	0.29 ± 0.01	mw	0.207		
	ADC	0.53 ± 0.03	0.53 ± 0.01	tt	0.857		

Significant *P* values are indicated in bold font.  *D* ^∗^, *D*, and ADC in 10^−3^ mm^2^/s. TBF and CBF are in mL/100 g/min. HGG: high grade gliomas, LGG: low grade gliomas, WM: contralateral semioval center. AUC: area under the curve. mw: Mann-Whitney *U* test, tt: Student's *t*-test.

**Table 3:** Histogram parameters of solid component in tumor from the ASL and MB-DWI from histogram analysis as well as ROC analysis in HGG and LGG (mean ± SD).

	Tumor	HGG	LGG	*P*	Test type	AUC	Cutoff
CBF	Mean (mL/100 g/min)	102.04 ± 45.55	62.26 ± 18.13	0.011	tt	0.783	
	Median (mL/100 g/min)	99.65 ± 42.08	62.59 ± 18.07	0.011	tt	0.801	
	Maximum (mL/100 g/min)	160.77 ± 80.72	91.27 ± 30.89	0.002	mw	**0.85**	**81**
	Minimum (mL/100 g/min)	54.08 ± 30.13	31.64 ± 8.96	0.022	tt	0.738	
	90th percentile (mL/100 g/min)	132.15 ± 68.18	78.91 ± 25.35	0.015	mw	0.787	
	Standard deviation (mL/100 g/min)	22.51 ± 16.07	13.15 ± 5.77	0.035	mw	0.755	
	Skewness	0.22 ± 0.32	−0.06 ± 0.25	0.024	tt	0.79	
	Kurtosis	2.92 ± 0.76	2.64 ± 0.44	0.649	mw	0.559	

*D* ^∗^	Mean (10^−3^ mm^2^/s)	5.1 ± 3.39	2.77 ± 0.69	0.002	mw	**0.857**	**3.5**
	Median (10^−3^ mm^2^/s)	3.54 ± 1.52	2.58 ± 0.33	0.002	mw	0.85	
	Maximum (10^−3^ mm^2^/s)	30.28 ± 21.91	9.38 ± 8.72	0.015	mw	0.79	
	Minimum (10^−3^ mm^2^/s)	0.84 ± 0.76	1.46 ± 0.77	0.03	mw	0.238	
	90th percentile (10^−3^ mm^2^/s)	9.86 ± 10.22	4.02 ± 1.46	0.006	mw	0.825	
	Standard deviation (10^−3^ mm^2^/s)	3.39 ± 3.35	13.8 ± 1.58	0.055	mw	0.734	
	Skewness	2.81 ± 1.93	1.66 ± 1.3	0.186	mw	0.664	
	Kurtosis	19.93 ± 20.46	10.04 ± 4.96	0.569	mw	0.573	

*D*	Mean (10^−3^ mm^2^/s)	0.69 ± 0.09	0.81 ± 0.12	0.013	tt	0.818	
	Median (10^−3^ mm^2^/s)	0.69 ± 0.09	0.82 ± 0.11	0.006	mw	**0.825**	**0.755**
	Maximum (10^−3^ mm^2^/s)	0.98 ± 0.19	1.07 ± 0.15	0.15	mw	0.678	
	Minimum (10^−3^ mm^2^/s)	0.29 ± 0.25	0.55 ± 0.25	0.039	tt	0.755	
	90th percentile (10^−3^ mm^2^/s)	0.83 ± 0.14	0.95 ± 0.11	0.029	tt	0.766	
	Standard deviation (10^−3^ mm^2^/s)	0.12 ± 0.05	0.12 ± 0.08	0.776	mw	0.465	
	Skewness	−0.45 ± 1.23	−0.42 ± 1.39	0.955	mw	0.49	
	Kurtosis	6.63 ± 5.09	5.12 ± 6.53	0.063	mw	0.273	

*f*	Mean (10^−3^ mm^2^/s)	0.4 ± 0.11	0.49 ± 0.1	0.039	tt	0.769	
	Median (10^−3^ mm^2^/s)	0.39 ± 0.12	0.49 ± 0.11	0.025	tt	**0.783**	**0.395**
	Maximum (10^−3^ mm^2^/s)	0.81 ± 0.18	0.84 ± 0.14	0.82	mw	0.528	
	Minimum (10^−3^ mm^2^/s)	0.14 ± 0.07	0.22 ± 0.12	0.042	tt	0.766	
	90th percentile (10^−3^ mm^2^/s)	0.57 ± 0.16	0.67 ± 0.15	0.14	tt	0.678	
	Standard deviation (10^−3^ mm^2^/s)	0.13 ± 0.05	0.14 ± 0.05	0.728	tt	0.528	
	Skewness	0.73 ± 0.69	0.29 ± 0.49	0.09	tt	0.297	
	Kurtosis	4.22 ± 2.83	2.89 ± 1.04	0.11	tt	0.301	

ADC	Mean (10^−3^ mm^2^/s)	0.85 ± 0.09	1.04 ± 0.13	0.001	tt	0.85	
	Median (10^−3^ mm^2^/s)	0.85 ± 0.11	1.07 ± 0.12	0.000	mw	**0.92**	**0.885**
	Maximum (10^−3^ mm^2^/s)	1.37 ± 0.29	1.42 ± 0.21	0.648	tt	0.58	
	Minimum (10^−3^ mm^2^/s)	0.43 ± 0.27	0.65 ± 0.28	0.054	tt	0.75	
	90th percentile (10^−3^ mm^2^/s)	1.07 ± 0.17	1.24 ± 0.16	0.019	tt	0.76	
	Standard deviation (10^−3^ mm^2^/s)	0.16 ± 0.07	0.17 ± 0.05	0.528	tt	0.6	
	Skewness	0.23 ± 1.5	−0.16 ± 0.89	0.457	tt	0.35	
	Kurtosis	6.67 ± 5.11	3.35 ± 1.95	0.04	tt	0.21	

Statistically significant values are presented in bold font. mw: Mann-Whitney *U* test, tt: Student's *t*-test.

LGG: low grade  gliomas, AUC: area under the curve.

**Table 4:** Histogram parameters of white matter from the ASL and MB-DWI from histogram analysis as well as ROC analysis in HGG and LGG (mean ± SD).

	White matter	HGG	LGG	Test type	*P*
CBF	Mean (mL/100 g/min)	25.25 ± 3.46	27.64 ± 8.21	mw	0.733
	Median (mL/100 g/min)	25.69 ± 5.15	27.33 ± 7.48	mw	0.649
	Maximum (mL/100 g/min)	38.62 ± 5.23	42.91 ± 12.49	mw	0.955
	Minimum (mL/100 g/min)	13.82 ± 5.44	15.91 ± 7.79	mw	0.459
	90th percentile (mL/100 g/min)	33.62 ± 4.75	35.18 ± 10.94	mw	0.459
	Standard deviation (mL/100 g/min)	6.77 ± 3.62	6.14 ± 2.39	mw	0.331
	Skewness	2.37 ± 7.86	2.32 ± 0.47	mw	0.776
	Kurtosis	2.38 ± 0.45	2.86 ± 0.55	mw	**0.03**

*D* ^∗^	Mean (10^−3^ mm^2^/s)	2.5 ± 0.18	2.52 ± 0.29	mw	0.691
	Median (10^−3^ mm^2^/s)	2.44 ± 0.18	2.48 ± 0.27	mw	0.91
	Maximum (10^−3^ mm^2^/s)	3.72 ± 0.62	3.79 ± 0.65	mw	0.733
	Minimum (10^−3^ mm^2^/s)	1.76 ± 0.16	1.82 ± 0.19	tt	0.429
	90th percentile (10^−3^ mm^2^/s)	2.99 ± 0.34	2.95 ± 0.26	tt	0.992
	Standard deviation (10^−3^ mm^2^/s)	0.42 ± 0.11	0.39 ± 0.09	tt	0.453
	Skewness	0.68 ± 0.36	0.75 ± 0.32	tt	0.627
	Kurtosis	3.49 ± 1.24	3.88 ± 1.2	mw	0.459

*D*	Mean (10^−3^ mm^2^/s)	0.41 ± 0.02	0.41 ± 0.02	tt	0.801
	Median (10^−3^ mm^2^/s)	0.4 ± 0.02	0.41 ± 0.02	tt	0.473
	Maximum (10^−3^ mm^2^/s)	0.47 ± 0.03	0.49 ± 0.04	tt	0.161
	Minimum (10^−3^ mm^2^/s)	0.35 ± 0.03	0.33 ± 0.02	tt	0.083
	90th percentile (10^−3^ mm^2^/s)	0.44 ± 0.03	0.45 ± 0.02	tt	0.385
	Standard deviation (10^−3^ mm^2^/s)	0.03 ± 0.01	0.04 ± 0.01	mw	0.252
	Skewness	0.11 ± 0.63	−0.11 ± 0.34	tt	0.308
	Kurtosis	2.56 ± 0.66	2.47 ± 0.41	tt	0.707

*f*	Mean (10^−3^ mm^2^/s)	0.32 ± 0.03	0.29 ± 0.01	mw	0.207
	Median (10^−3^ mm^2^/s)	0.3 ± 0.02	0.29 ± 0.01	mw	0.608
	Maximum (10^−3^ mm^2^/s)	0.4 ± 0.08	0.37 ± 0.04	mw	0.331
	Minimum (10^−3^ mm^2^/s)	0.25 ± 0.02	0.26 ± 0.01	mw	0.82
	90th percentile (10^−3^ mm^2^/s)	0.37 ± 0.08	0.33 ± 0.03	mw	0.15
	Standard deviation (10^−3^ mm^2^/s)	0.04 ± 0.03	0.02 ± 0.01	mw	0.207
	Skewness	0.54 ± 0.47	0.47 ± 0.76	tt	0.785
	Kurtosis	3.07 ± 1.41	3.41 ± 0.84	mw	0.106

ADC	Mean (10^−3^ mm^2^/s)	0.53 ± 0.03	0.53 ± 0.01	tt	0.857
	Median (10^−3^ mm^2^/s)	0.53 ± 0.03	0.53 ± 0.02	tt	0.988
	Maximum (10^−3^ mm^2^/s)	0.64 ± 0.08	0.62 ± 0.05	tt	0.64
	Minimum (10^−3^ mm^2^/s)	0.46 ± 0.02	0.45 ± 0.02	tt	0.293
	90th percentile (10^−3^ mm^2^/s)	0.59 ± 0.08	0.58 ± 0.05	tt	0.826
	Standard deviation (10^−3^ mm^2^/s)	0.04 ± 0.02	0.04 ± 0.02	mw	0.82
	Skewness	0.45 ± 0.8	0.13 ± 0.57	tt	0.278
	Kurtosis	3.28 ± 1.47	2.78 ± 0.74	mw	0.392

Statistically significant values are presented in bold font. mw: Mann-Whitney *U* test, tt: Student's *t*-test. LGG: low  grade  gliomas, WM: contralateral semioval center. AUC: area under the curve.

## References

[1] Le Bihan D., Breton E., Lallemand D., Aubin M.-L., Vignaud J., Laval-Jeantet M. (1988). Separation of diffusion and perfusion in intravoxel incoherent motion MR imaging. *Radiology*.

[2] Maier S. E., Bogner P., Bajzik G. (2001). Normal brain and brain tumor: multicomponent apparent diffusion coefficient line scan imaging. *Radiology*.

[3] Ichikawa S., Motosugi U., Ichikawa T., Sano K., Morisaka H., Araki T. (2013). Intravoxel incoherent motion imaging of focal hepatic lesions. *Journal of Magnetic Resonance Imaging*.

[4] Eckerbom P., Hansell P., Bjerner T., Palm F., Weis J., Liss P. (2013). Intravoxel incoherent motion MR imaging of the kidney: pilot study. *Advances in Experimental Medicine and Biology*.

[5] Pang Y., Turkbey B., Bernardo M. (2013). Intravoxel incoherent motion MR imaging for prostate cancer: an evaluation of perfusion fraction and diffusion coefficient derived from different *b*-value combinations. *Magnetic Resonance in Medicine*.

[6] Lu Y., Jansen J. F. A., Stambuk H. E. (2013). Comparing primary tumors and metastatic nodes in head and neck cancer using intravoxel incoherent motion imaging: a preliminary experience. *Journal of Computer Assisted Tomography*.

[7] Bisdas S., Koh T. S., Roder C. (2013). Intravoxel incoherent motion diffusion-weighted MR imaging of gliomas: feasibility of the method and initial results. *Neuroradiology*.

[8] Federau C., O'Brien K., Meuli R., Hagmann P., Maeder P. (2014). Measuring brain perfusion with intravoxel incoherent motion (IVIM): initial clinical experience. *Journal of Magnetic Resonance Imaging*.

[9] Hu Y. C., Yan L. F., Wu L. (2014). Intravoxel incoherent motion diffusion-weighted MR imaging of gliomas: efficacy in preoperative grading. *Scientific Reports*.

[10] Mulkern R. V., Vajapeyam S., Robertson R. L., Caruso P. A., Rivkin M. J., Maier S. E. (2001). Biexponential apparent diffusion coefficient parametrization in adult vs newborn brain. *Magnetic Resonance Imaging*.

[11] Lemke A., Stieltjes B., Schad L. R., Laun F. B. (2011). Toward an optimal distribution of b values for intravoxel incoherent motion imaging. *Magnetic Resonance Imaging*.

[12] Hirai T., Kitajima M., Nakamura H. (2011). Quantitative blood flow measurements in gliomas using arterial spin-labeling at 3T: intermodality agreement and inter- and intraobserver reproducibility study. *American Journal of Neuroradiology*.

[13] Wong A. M., Yan F.-X., Liu H.-L. (2014). Comparison of three-dimensional pseudo-continuous arterial spin labeling perfusion imaging with gradient-echo and spin-echo dynamic susceptibility contrast MRI. *Journal of Magnetic Resonance Imaging*.

[14] Järnum H., Steffensen E. G., Knutsson L. (2010). Perfusion MRI of brain tumours: a comparative study of pseudo-continuous arterial spin labelling and dynamic susceptibility contrast imaging. *Neuroradiology*.

[15] Falk A., Fahlström M., Rostrup E. (2014). Discrimination between glioma grades II and III in suspected low-grade gliomas using dynamic contrast-enhanced and dynamic susceptibility contrast perfusion MR imaging: a histogram analysis approach. *Neuroradiology*.

[16] Cohen Y., Assaf Y. (2002). High *b*-value q-space analyzed diffusion-weighted MRS and MRI in neuronal tissues—a technical review. *NMR in Biomedicine*.

[17] Bennett K. M., Schmainda K. M., Bennett R., Rowe D. B., Lu H., Hyde J. S. (2003). Characterization of continuously distributed cortical water diffusion rates with a stretched-exponential model. *Magnetic Resonance in Medicine*.

[18] Federau C., Maeder P., O'Brien K., Browaeys P., Meuli R., Hagmann P. (2012). Quantitative measurement of brain perfusion with intravoxel incoherent motion MR imaging. *Radiology*.

[19] Wirestam R., Borg M., Brockstedt S., Lindgren A., Holtås S., Ståhlberg F. (2001). Perfusion-related parameters in intravoxel incoherent motion MR imaging compared with CBV and CBF measured by dynamic susceptibility-contrast MR technique. *Acta Radiologica*.

[20] Bennett K. M., Hyde J. S., Schmainda K. M. (2006). Water diffusion heterogeneity index in the human brain is insensitive to the orientation of applied magnetic field gradients. *Magnetic Resonance in Medicine*.

[21] Guiu B., Petit J.-M., Capitan V. (2012). Intravoxel incoherent motion diffusion-weighted imaging in nonalcoholic fatty liver disease: a 3.0-T MR study. *Radiology*.

[22] Lemke A., Laun F. B., Simon D., Stieltjes B., Schad L. R. (2010). An in vivo verification of the intravoxel incoherent motion effect in diffusion-weighted imaging of the abdomen. *Magnetic Resonance in Medicine*.

[23] Yingying H., Ye W., Sheng X. (2013). Impact of head position on cerebral blood flow measured by 3D pseudo-continuous arterial spine labeling. *Chinese Journal of Medical Imaging Technology*.

[24] Wu B., Lou X., Wu X., Ma L. (2014). Intra- and interscanner reliability and reproducibility of 3D whole-brain pseudo-continuous arterial spin-labeling MR perfusion at 3T. *Journal of Magnetic Resonance Imaging*.

[25] Knutsson L., Ståhlberg F., Wirestam R. (2010). Absolute quantification of perfusion using dynamic susceptibility contrast MRI: pitfalls and possibilities. *Magnetic Resonance Materials in Physics, Biology and Medicine*.

[26] Rosen B. R., Belliveau J. W., Vevea J. M., Brady T. J. (1990). Perfusion imaging with NMR contrast agents. *Magnetic Resonance in Medicine*.

[27] Vilringer A., Rosen B. R., Belliveau J. W. (1988). Dynamic imaging with lanthanide chelates in normal brain: contrast due to magnetic susceptibility effects. *Magnetic Resonance in Medicine*.

[28] Henkelman R. M. (1990). Does IVIM measure classical perfusion?. *Magnetic Resonance in Medicine*.

[29] Le Bihan D., Turner R. (1992). The capillary network: a link between IVIM and classical perfusion. *Magnetic Resonance in Medicine*.

